# MRI-based quantification of intratumoral heterogeneity for intrahepatic mass-forming cholangiocarcinoma grading: a multicenter study

**DOI:** 10.1186/s13244-025-01985-9

**Published:** 2025-05-14

**Authors:** Liyong Zhuo, Wenjing Chen, Lihong Xing, Xiaomeng Li, Zijun Song, Jinghui Dong, Yanyan Zhang, Hongjun Li, Jingjing Cui, Yuxiao Han, Jiawei Hao, Jianing Wang, Xiaoping Yin, Caiying Li

**Affiliations:** 1 Department of Medical Imaging, The Second Hospital of Hebei Medical University, Shijiazhuang, People’s Republic of China; 2 Department of Radiology, Affiliated Hospital of Hebei University, Baoding, People’s Republic of China; 3 Department of Research and Development, United Imaging Intelligence (Beijing) Co., Ltd., Beijing, People’s Republic of China; 4 Department of Critical Care Medicine, Baoding First Central Hospital, Baoding, People’s Republic of China; 5 Department of Radiology, The Fifth Medical Center of Chinese PLA General Hospital, Beijing, People’s Republic of China; 6 Department of Radiology, Beijing You’an Hospital, Beijing, People’s Republic of China

**Keywords:** Cholangiocarcinoma, Neoplasm grading, Magnetic resonance imaging, Radiomics, Habitat imaging

## Abstract

**Objective:**

This study aimed to develop a quantitative approach to measure intratumor heterogeneity (ITH) using MRI scans and predict the pathological grading of intrahepatic mass-forming cholangiocarcinoma (IMCC).

**Methods:**

Preoperative MRI scans from IMCC patients were retrospectively obtained from five academic medical centers, covering the period from March 2018 to April 2024. Radiomic features were extracted from the whole tumor and its subregions, which were segmented using K-means clustering. An ITH index was derived from a habitat model integrating output probabilities of the subregions-based models. Significant variables from clinical laboratory-imaging features, radiomics, and the habitat model were integrated into a predictive model, and its performance was evaluated using the area under the receiver operating characteristic curve (AUC).

**Results:**

The final training and internal validation datasets included 197 patients (median age, 59 years [IQR, 52–65 years]); the external validation dataset included 43 patients (median age, 58.5 years [IQR, 52.25–69.75 years]). The habitat model achieved AUCs of 0.847 (95% CI: 0.783, 0.911) in the training set and 0.753 (95% CI: 0.595, 0.911) in the internal validation set. Furthermore, the combined model, integrating imaging variables, the habitat model, and radiomics model, demonstrated improved predictive performance, with AUCs of 0.895 (95% CI: 0.845, 0.944) in the training dataset, 0.790 (95% CI: 0.65, 0.931) in the internal validation dataset, and 0.815 (95% CI: 0.68, 0.951) in the external validation dataset.

**Conclusion:**

The combined model based on MRI-derived quantification of ITH, along with clinical, laboratory, radiological, and radiomic features, showed good performance in predicting IMCC grading.

**Critical relevance statement:**

This model, integrating MRI-derived intrahepatic mass-forming cholangiocarcinoma (IMCC) classification metrics with quantitative radiomic analysis of intratumor heterogeneity (ITH), demonstrates enhanced accuracy in tumor grade prediction, advancing risk stratification for clinical decision-making in IMCC management.

**Key Points:**

- Grading of intrahepatic mass-forming cholangiocarcinoma (IMCC) is important for risk stratification, clinical decision-making, and personalized therapeutic optimization.
- Quantitative intratumor heterogeneity can accurately predict the pathological grading of IMCC.
- This combined model provides higher diagnostic accuracy.

**Graphical Abstract:**

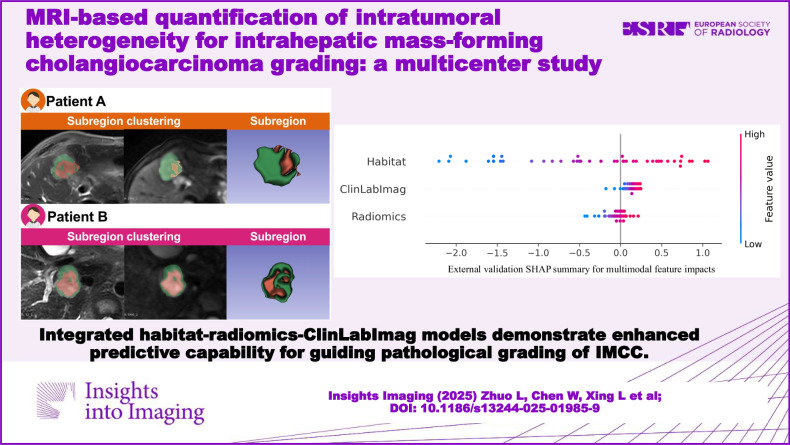

## Introduction

Intrahepatic cholangiocarcinoma (ICC) is the second most prevalent type of primary liver cancer, following hepatocellular carcinoma [1]. Intrahepatic mass-forming cholangiocarcinoma (IMCC) is the most frequent and exhibits the poorest prognosis, accounting for approximately 60% of ICC cases [2, 3].

Tumor grading is a critical prognostic indicator for ICC, closely associated with tumor recurrence rates [4] and significantly influencing patient survival [5, 6]. ICC with a lower pathological grade is typically associated with a more active immunosuppressive microenvironment [7, 8], enhanced angiogenesis [8], and greater intratumoral heterogeneity [9]. However, traditional pathological assessment of IMCC relies on invasive histopathological examinations, which are subject to sampling bias, time-consuming, and limit their clinical applicability [10, 11]. As a result, there is an increasing demand for a reliable, non-invasive imaging technique to predict tumor grade preoperatively.

Habitat imaging, an innovative non-invasive imaging technique, offers a new perspective for in-depth tumor analysis by segmenting the tumor into subregions with distinct biological characteristics, referred to as “habitats” [12, 13]. The spatial distribution of these habitats can be qualitatively visualized, while quantitative analysis can be performed by calculating the volume proportion of each habitat, the proportional differences between them [12, 13], and the intratumoral heterogeneity (ITH) index [14, 15]. Habitat imaging is highly sensitive in capturing biological differences within various regions of a tumor, monitoring dynamic changes in tumor composition, and providing a comprehensive reflection of ITH. This technique is valuable for accurate diagnosis [12], evaluation of treatment response [14], and prediction of pathological indicators [16] in malignant tumors. Thus, a quantitative measurement of ITH may act as a valuable biomarker for predicting the pathological grade of IMCC.

In recent years, diffusion-weighted imaging (DWI) and T2-weighted imaging (T2WI) have shown great potential in characterizing IMCC. DWI provides metrics that quantify various tissue characteristics, such as cellularity and tissue heterogeneity [17, 18], with its signal characteristics differing across various degrees of IMCC differentiation. On the other hand, T2WI supplements DWI by offering high-resolution images of the tumor anatomy and surrounding structures [19]. By combining the microstructural changes and differences in cellular density captured by DWI with the detailed anatomical and compositional information provided by T2WI, a more comprehensive assessment of ITH can be achieved [20].

This study aims to develop a quantitative measurement of ITH through habitat Imaging based on MRI to predict the pathological grade of IMCC.

## Materials and methods

**Fig. 1:**
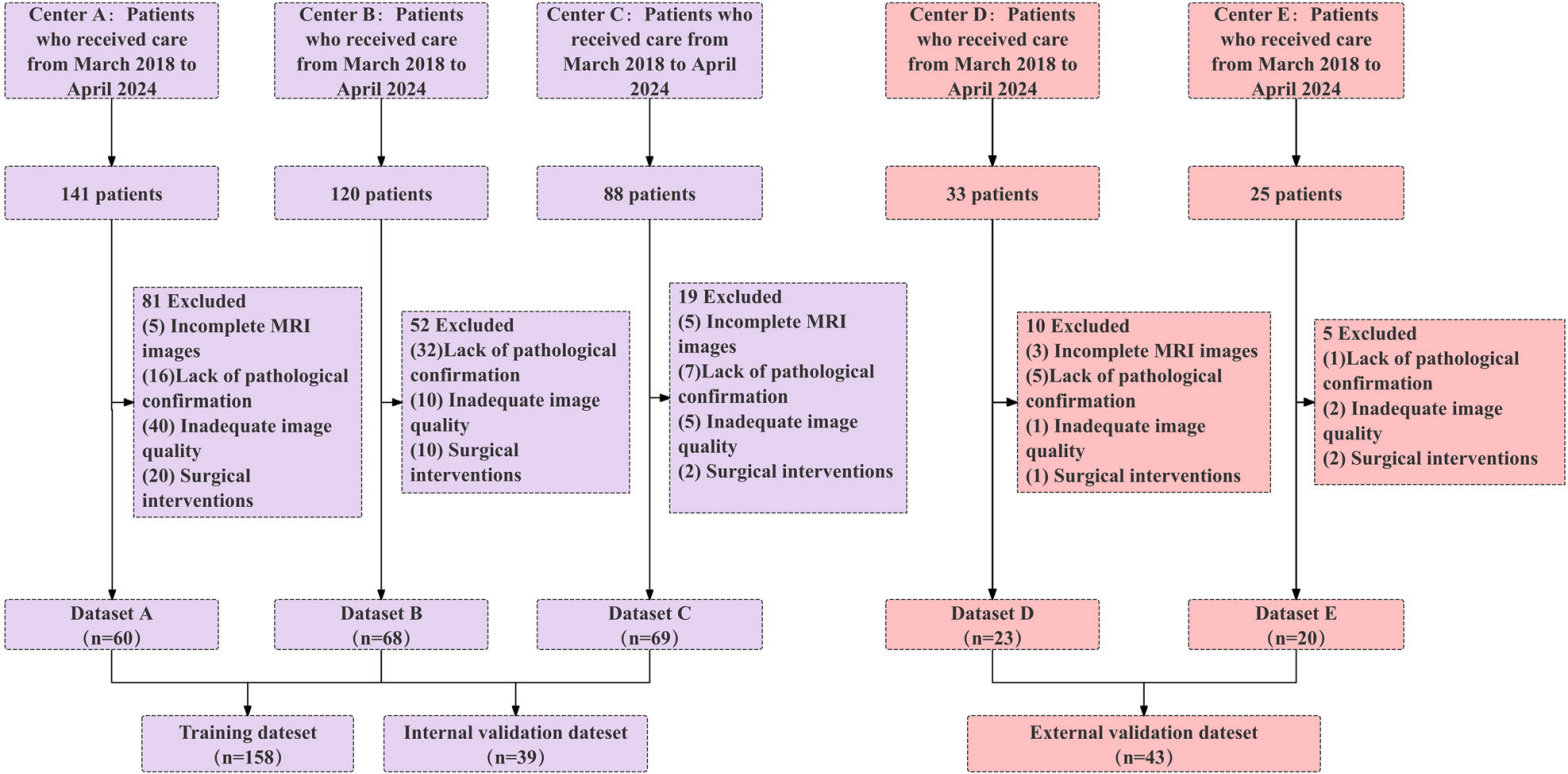
Flowchart of the patient inclusion and exclusion process

This study adhered to the Declaration of Helsinki and was approved by the ethics committees of the participating medical centers (HDFYLL-KY-2024-021), with a waiver of written informed consent for all participants. The study dataset included patients from three distinct regional academic medical centers, with external validation conducted using independent datasets from two medical centers in separate regions (Fig. 1). The inclusion criteria for all participants were as follows: (1) All patients had pathologically confirmed IMCC. (2) All patients underwent preoperative MRI. The exclusion criteria were: (1) Patients who had received systemic or locoregional therapies (e.g., chemotherapy, radiotherapy, or ablation) prior to surgical resection. (2) Patients with a prolonged interval between MRI and surgical resection or biopsy (exceeding 2 weeks). (3) Patients with poor-quality MRI images due to severe artifacts, insufficient signal-to-noise ratio, or incomplete coverage of the tumor region.

Patients were randomly allocated to training and internal validation cohorts in an 8:2 ratio using stratified sampling based on pathological grade to ensure proportional distribution of high-grade and low-grade IMCC cases in both cohorts.

### Histopathologic assessment

All surgical specimens underwent histological staining and pathological review by two experienced pathologists across five institutions, with central readings conducted blind to MRI findings. After completing their independent assessments, they reviewed each other’s findings, and in cases of discrepancies, consensus was reached through discussion. All IMCC cases were classified per WHO criteria [21] into low-, medium-, and high-grade categories, with medium- and high-grade tumors consolidated into a single high-grade cohort for analysis due to their prognostic equivalence [22] and limited medium-grade sample size.

### MRI procedure and image preprocessing

The MRI acquisition parameters are detailed in Table S1. Image preprocessing involved N4 bias field correction [23], resampling to isotropic voxels of 1 mm³ using B-spline interpolation, and histogram standardization of intensity values in the MRI scans [24]. In this study, image registration between DWI and T2WI was achieved through a combination of affine and deformable transformations, with mutual information used as the optimization metric.

### Image analysis

All images were independently reviewed by two radiologists (radiologist A with 10 years of experience and radiologist B with 5 years of experience in abdominal MRI), respectively, using a picture archiving and communication system (Tianjin Technology Group, Zhongguancun Fengtai Science Park, Beijing, China). Both radiologists were blinded to the patients’ clinical and histopathological information.

The “targetoid sign” was described as having a relatively hyperintense peripheral ring or a relatively hyperintense central area. Tumor thrombus refers to the growth or extension of a tumor into adjacent blood vessels. The vascular traversal sign is described as the extension of blood vessels into the interior of the tumor. Intrahepatic duct dilatation was defined as abrupt caliber changes in bile ducts adjacent to the tumor, appearing hyperintense on T2WI [25]. Hepatic capsular retraction was defined as inward distortion of the liver surface adjacent to the tumor mass. Enlarged lymph nodes were characterized by a short-axis diameter of 1 cm or greater [26].

After completing the independent evaluations, inter-observer agreement was quantified using intraclass correlation coefficients for continuous variables and Cohen’s kappa (κ) for categorical variables. The intraclass correlation coefficient values ranged from 0.82 to 0.93 for quantitative imaging features, while κ values were 0.75–0.88 for qualitative assessments, indicating substantial to excellent agreement. For cases with discrepancies, the two reviewers discussed and reached a consensus on the final interpretation.

### Tumor segmentation and habitat subregion clustering

The three-dimensional tumor regions were manually delineated by two radiologists (radiologist A with 10 years of experience and radiologist B with 5 years of experience in abdominal MRI) on the DWI images (b = 800 s/mm²) using ITK-SNAP software (version 4.0.0; http://www.itksnap.org/pmwiki/pmwiki.php). The final volumes of interest (VOIs) were determined through consensus between the two radiologists and validated by a third radiologist (L.X.), who has over 15 years of experience. The delineation of VOIs was performed using a free-hand drawing method, followed by automatic smoothing and interpolation of contours using ITK-SNAP’s built-in algorithms to ensure spatial continuity and volumetric accuracy.

In this study, we employed the K-means algorithm to perform clustering analysis based on the grayscale features of the tumor region on DWI and T2WI images. The K-means algorithm automatically classifies data by minimizing the distance between each sample and the centroid within a cluster. To determine the optimal number of clusters (K), we utilized the elbow method as a decision-making aid.

**Fig. 2:**
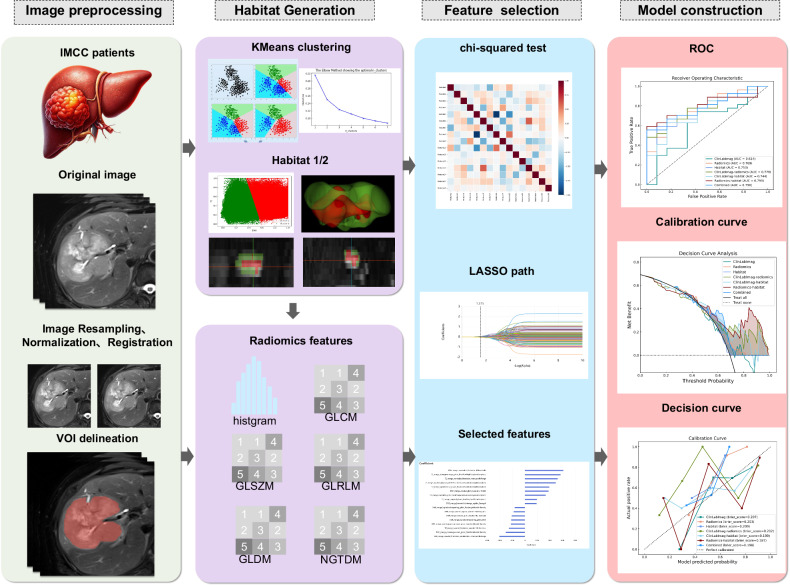
Radiomics and habitat clustering pipeline of the study. IMCC, intrahepatic mass-forming cholangiocarcinoma; VOI, volume of interest; GLCM, gray-level co-occurrence matrix; GLSZM, gray-level size zone matrix; GLRLM, gray-level run-length matrix; GLDM, gray-level dependence matrix; NGTDM, neighboring gray-tone difference matrix; LASSO, least absolute shrinkage and selection operator; ROC, receiver operating characteristic

Initiating with K = 1, we incrementally raised K, executing K-means and computing sum of squared errors (SSE) for each, then plotted SSE versus K. SSE declined with increasing K, but decelerated markedly at an “elbow” point. This K value is considered the optimal number of clusters. In this study, the optimal K value was found to be 2 (Fig. 2), resulting in two subregions for both T2WI and DWI.

### Radiomics feature extraction

Radiomic feature extraction was performed using the Pyradiomics V3.0 [27] tool integrated into the uAI Research Portal V1.1 (Shanghai United Imaging Intelligence, Co., Ltd.) [28]. A total of 1904 radiomic features were extracted from T2WI, DWI, and four subregions, respectively. This process involved the application of 16 different filters (supplementary material), which captured a range of features, including first-order statistical parameters (*n* = 378), morphological parameters (*n* = 14), gray-level co-occurrence matrix (GLCM) parameters (*n* = 441), gray-level run-length matrix (GLRLM) parameters (*n* = 336), gray-level size zone matrix (GLSZM) parameters (*n* = 336), gray-level dependence matrix (GLDM) parameters (*n* = 294), and neighboring gray-tone difference matrix (NGTDM) parameters (*n* = 105).

### Feature selection and model construction

For the habitat model, the features extracted from the four subregions of T2WI and DWI (DWI_habitat1_, DWI_habitat2_, T2WI_habitat1_, T2WI_habitat2_) were first subjected to chi-square tests. Features with a *p*-value less than 0.05 were then selected, followed by the application of the least absolute shrinkage and selection operator (LASSO) method. LASSO was chosen for its sparsity, stability, and interpretability, helping to reduce feature redundancy and eliminate high correlations. Second, logistic regression models were developed for each of the four subregions, and the output probabilities from these models were used to generate an intratumoral heterogeneity (ITH) index at the patient level. To confirm that the ITH index could effectively predict the pathological grade of IMCC, we conducted random combinations of the four ITH scores. The model with the highest diagnostic performance in the internal validation set was selected as the final habitat model.

For traditional radiomics features extracted from both T2WI and DWI sequences, the same chi-square test and LASSO feature selection methods were applied. Radiomic features with a *p*-value less than 0.05 were ultimately chosen for model development.

Clinical, laboratory, and imaging features showing significant association (*p* < 0.05) with pathological grade in univariable logistic regression (LR) were entered into multivariable analysis. Only variables retaining significance (*p* < 0.05) in the multivariable model were included in the final ClinLabImag model. Finally, seven predictive models were developed using LR in Python, incorporating the following features: (1) Clinical, laboratory, and imaging features (ClinLabImag model); (2) Traditional radiomics features (Radiomics model); (3) Habitat imaging features (Habitat model); (4) Clinical, laboratory, imaging, and traditional radiomics features (ClinLabImag-Radiomics model); (5) Clinical, laboratory, imaging, and habitat imaging features (ClinLabImag-Habitat model); (6) Traditional radiomics and habitat imaging features (Radiomics-Habitat model); (7) Clinical, laboratory, imaging, traditional radiomics, and habitat imaging features (Combined model).

### Statistical analysis

Continuous variables are presented as medians with interquartile ranges (IQRs), while categorical variables are expressed as frequencies and percentages. To compare patient characteristics across the training, internal validation, and external validation datasets, either the Fisher exact test, Pearson χ² test, or Mann–Whitney U-test was applied, depending on the data type. Additionally, univariable and multivariable logistic regression analyses were performed to assess differences in patient characteristics across these datasets.

The models’ predictive performance for IMCC grading was assessed by calculating the area under the receiver operating characteristic curve (AUC) with 95% confidence intervals (CI). In addition, key metrics including sensitivity, specificity, accuracy, and precision were calculated to further assess model efficacy. To analyze the contribution of individual features to the model’s predictions, Shapley additive explanations analysis [27] (SHAP) was conducted. This approach quantified the impact of clinical, laboratory, imaging, habitat, and radiomics features on the model’s outcomes.

Statistical analyses were conducted utilizing R (version 3.6.0; The R Foundation). Two-tailed *p* < 0.05 was considered indicative of a statistically significant difference.

## Results

### Patient characteristics

A total of 407 patients were initially collected from five centers (Fig. 1). The training and internal validation dataset consisted of 197 IMCC patients from Center A (*n* = 60), Center B (*n* = 68), and Center C (*n* = 69) with a median age of 59 years (IQR: 52–65 years). The external validation dataset included 43 patients with IMCC from Center D (*n* = 23) and Center F (*n* = 20), with a median age of 58.5 years (IQR: 52.25–69.75 years). There were no statistically significant differences in clinical, laboratory, or imaging characteristics between the training, internal validation, and external validation datasets. Detailed patient characteristics are provided in Table 1.

**Table 1:** Comparative analysis of clinical, laboratory, and imaging features in the training, internal and external validation cohorts

Characteristic	Training cohorts (*n* = 158)			Internal validation cohorts (*n* = 39)			External validation cohorts (*n* = 43)			*p*-inter value
	Low-grade (*n* = 51)	High-grade (*n* = 107)	*p*-intra value	Low-grade (*n* = 12)	High-grade (*n* = 27)	*p*-intra value	Low-grade (*n* = 14)	High-grade (*n* = 29)	*p*-intra value	
Age (year)	61.000 [55.000, 67.500]	59.000 [51.500, 65.000]	0.108	57.000 [51.750, 68.250]	59.000 [54.000, 64.500]	0.939	64.000 [54.000, 74.000]	56.000 [51.000, 68.000]	0.253	0.943
Sex			0.251			0.168			0.916	0.156
Male	13 (25.490)	37 (34.579)		3 (25.000)	14 (51.852)		7 (50.000)	14 (48.276)		
Female	38 (74.510)	70 (65.421)		9 (75.000)	13 (48.148)		7 (50.000)	15 (51.724)		
CA19-9			0.064			0.896			0.810	0.097
≤ 39	14 (27.451)	17 (15.888)		2 (16.667)	5 (18.519)		11 (78.571)	19 (65.517)		
> 39	18 (35.294)	30 (28.037)		5 (41.667)	8 (29.630)		3 (21.429)	9 (31.034)		
CEA			0.069			0.657			0.060	0.097
≤ 4.7	19 (37.255)	31 (28.972)		5 (41.667)	7 (25.926)		5 (35.714)	20 (68.966)		
> 4.7	13 (25.490)	16 (14.953)		2 (16.667)	6 (22.222)		9 (64.286)	8 (27.586)		
AFP			0.012*			0.337			0.590	0.097
≤ 7	25 (49.020)	39 (36.449)		6 (50.000)	12 (44.444)		13 (92.857)	22 (75.862)		
> 7	12 (23.529)	13 (12.150)		1 (8.333)	0 (0.000)		1 (7.143)	6 (20.690)		
CA12-5			0.192			0.884			0.587	0.264
≤ 35	7 (13.725)	7 (6.542)		2 (16.667)	3 (11.111)		11 (78.571)	18 (62.069)		
> 35	11 (21.569)	18 (16.822)		3 (25.000)	6 (22.222)		2 (14.286)	9 (31.034)		
Lesion location			0.328			0.013*			0.036*	0.395
Left lobe	8 (15.686)	22 (20.561)		0 (0.000)	11 (40.741)		8 (57.143)	22 (75.862)		
Right lobe	23 (45.098)	56 (52.336)		7 (58.333)	7 (25.926)		6 (42.857)	7 (24.138)		
Both	20 (39.216)	29 (27.103)		5 (41.667)	9 (33.333)		0 (0.000)	0 (0.000)		
Tumor margin			0.246			0.291			0.042*	0.441
Smooth	30 (58.824)	73 (68.224)		9 (75.000)	14 (51.852)		2 (14.286)	15 (51.724)		
Infiltrative	21 (41.176)	34 (31.776)		3 (25.000)	13 (48.148)		12 (85.715)	14 (48.276)		
T2WI tumor boundary			0.558			1.000			0.180	0.223
Clear	28 (54.902)	64 (59.813)		9 (75.000)	21 (77.778)		1 (7.143)	7 (24.138)		
Blurry	23 (45.098)	43 (40.187)		3 (25.000)	6 (22.222)		13 (92.857)	22 (75.862)		
Bile duct dilatation			0.436			0.731			0.329	0.223
Absent	31 (60.784)	58 (54.206)		5 (41.667)	14 (51.852)		7 (50.000)	19 (65.517)		
Present	20 (39.216)	49 (45.794)		7 (58.333)	13 (48.148)		7 (50.000)	10 (34.483)		
Hepatic capsule retraction			0.898			0.486			0.590	0.223
Absent	32 (62.745)	66 (61.682)		7 (58.333)	19 (70.370)		13 (92.857)	28 (96.552)		
Present	19 (37.255)	41 (38.318)		5 (41.667)	8 (29.630)		1 (7.143)	1 (3.448)		
Intrahepatic bile duct stones			0.707			NaN			0.706	0.368
Absent	49 (96.078)	104 (97.196)		12 (100.000)	27 (100.000)		12 (85.714)	26 (89.655)		
Present	2 (3.922)	3 (2.804)		0 (0.000)	0 (0.000)		2 (14.286)	3 (10.345)		
Tumor thrombus			0.165			0.041*			NaN	0.223
Absent	27 (52.941)	69 (64.486)		4 (33.333)	19 (70.370)		14 (100.000)	29 (100.000)		
Present	24 (47.059)	38 (35.514)		8 (66.667)	8 (29.630)		0 (0.000)	0 (0.000)		
Vascular traversal sign			0.496			0.287			NaN	0.223
Absent	34 (66.667)	77 (71.963)		6 (50.000)	19 (70.370)		14 (100.000)	29 (100.000)		
Present	17 (33.333)	30 (28.037)		6 (50.000)	8 (29.630)		0 (0.000)	0 (0.000)		
Enlarged lymph nodes			0.687			0.221			0.191	0.223
Absent	41 (80.392)	83 (77.570)		8 (66.667)	23 (85.185)		12 (85.714)	28 (96.552)		
Present	10 (19.608)	24 (22.430)		4 (33.333)	4 (14.815)		2 (14.286)	1 (3.448)		
T2WI signal pattern			0.490			1.000			NaN	0.223
Targetoid sign	37 (72.549)	83 (77.570)		11 (91.667)	23 (85.185)		14 (100.000)	29 (100.000)		
No-targetoid sign	14 (27.451)	24 (22.430)		1 (8.333)	4 (14.815)		0 (0.000)	0 (0.000)		
DWI signal pattern			0.460			1.000			0.592	0.424
Targetoid sign	35 (68.627)	67 (62.617)		10 (83.333)	23 (85.185)		3 (21.429)	3 (10.345)		
No-targetoid sign	16 (31.373)	40 (37.383)		2 (16.667)	4 (14.815)		11 (78.571)	26 (89.655)		

Categorical variables are expressed as frequency with percentages in parentheses. Qualitative variables are analyzed using Pearson’s χ2 test or Fisher’s exact test as appropriate, and quantitative variables are analyzed using the Mann–Whitney U-test. *p*-intra is the result of univariate analyses between the high-grade and low-grade groups; *p*-inter value represents the comparisons of characteristics between training, internal validation and external validation sets. Medium-grade and high-grade tumors were combined as high-grade for comparative analysis. Thresholds for positivity: CA19-9 > 39 U/mL, CEA > 4.7 ng/mL, AFP > 7 ng/mL, CA12-5 > 35 U/mL

*CA19-9* carbohydrate antigen 19-9, *CEA* carcinoembryonic antigen, *AFP* α-fetoprotein, *CA12-5* carbohydrate antigen 12-5, *DWI* diffusion-weighted imaging, *T2WI* T2-weighted imaging

* *p* < 0.05

### Variables associated with pathological grading in the training dataset

As presented in Table 2, univariable logistic regression analysis of clinical, laboratory, and imaging parameters revealed that the T2WI signal pattern (odds ratio (OR), 1.701 [95% CI: 1.188, 2.436]; *p* = 0.004) and tumor margin (OR, 0.651 [95% CI: 0.467, 0.908]; *p* = 0.012) were significantly associated with pathological grading in the training dataset. Similarly, multivariable logistic regression analysis demonstrated that the T2WI signal pattern (OR, 1.628 [95% CI: 1.074, 2.467]; *p* = 0.022) and tumor margin (OR, 0.613 [95% CI: 0.400, 0.940]; *p* = 0.025) remained significantly associated with pathological grading in the training dataset.

**Table 2:** Univariate and multivariable logistic regression analysis of the relationship between pathological grading and patient characteristics

Variables	Univariate analysis			Multivariable analysis		
	Odds ratio	95% CI	*p*-value	Odds ratio	95% CI	*p*-value
Age (year)	0.725	0.512–1.025	0.069			
Sex						
Female vs. male	0.817	0.578–1.155	0.252			
CA19-9						
> 39 vs. ≤ 39	1.063	0.762–1.484	0.717			
CEA						
> 4.7 vs. ≤ 4.7	0.968	0.694–1.351	0.849			
AFP						
> 7 vs. ≤ 7	1.017	0.728–1.420	0.921			
CA12-5						
> 35 vs. ≤ 35	1.049	0.751–1.465	0.780			
Lesion location						
Left lobe vs. right lobe vs. both	0.780	0.555–1.097	0.154			
Tumor margin						
Infiltrative vs. smooth	0.651	0.467–0.908	0.012*	0.613	0.400–0.940	0.025*
T2WI tumor boundary						
Blurry vs. clear	0.906	0.650–1.262	0.559			
Bile duct dilatation						
Present vs. absent	1.143	0.816–1.601	0.436			
Hepatic capsule retraction						
Present vs. absent	1.022	0.732–1.428	0.898			
Intrahepatic bile duct stones						
Present vs. absent	0.941	0.684–1.294	0.709			
Vascular involvement						
Present vs. absent	0.792	0.569–1.102	0.166			
Vascular traversal sign						
Present vs. absent	0.892	0.642–1.239	0.496			
Enlarged lymph nodes						
Present vs. absent	1.072	0.763–1.507	0.687			
DWI signal pattern						
Targetoid sign vs. no-targetoid sign	1.029	0.739–1.434	0.865			
T2WI signal pattern						
Targetoid sign vs. no-targetoid sign	1.701	1.188–2.436	0.004*	1.628	1.074–2.467	0.022*

Thresholds for positivity: CA19-9 > 39 U/mL, CEA > 4.7 ng/mL, AFP > 7 ng/mL, CA12-5 > 35 U/mL

*CI* confidence intervals, *CA19-9* carbohydrate antigen 19-9, *CEA* carcinoembryonic antigen, *AFP* α-fetoprotein, *CA12-5* carbohydrate antigen 12-5, *DWI* diffusion-weighted imaging, *T2WI* T2-weighted imaging

* *p* < 0.05

A total of 1904 features were extracted from each of the six sequences, including T2WI, DWI, and the four subregions. Ultimately, the following number of radiomic features were selected for tumor pathological grading prediction: 11 from DWI_habitat1_, 7 from DWI_habitat2_, 11 from T2WI_habitat1_, 12 from T2WI_habitat2_, and 16 from the Radiomics model.

### Habitat models for pathological grading in the training dataset

**Fig. 3:**
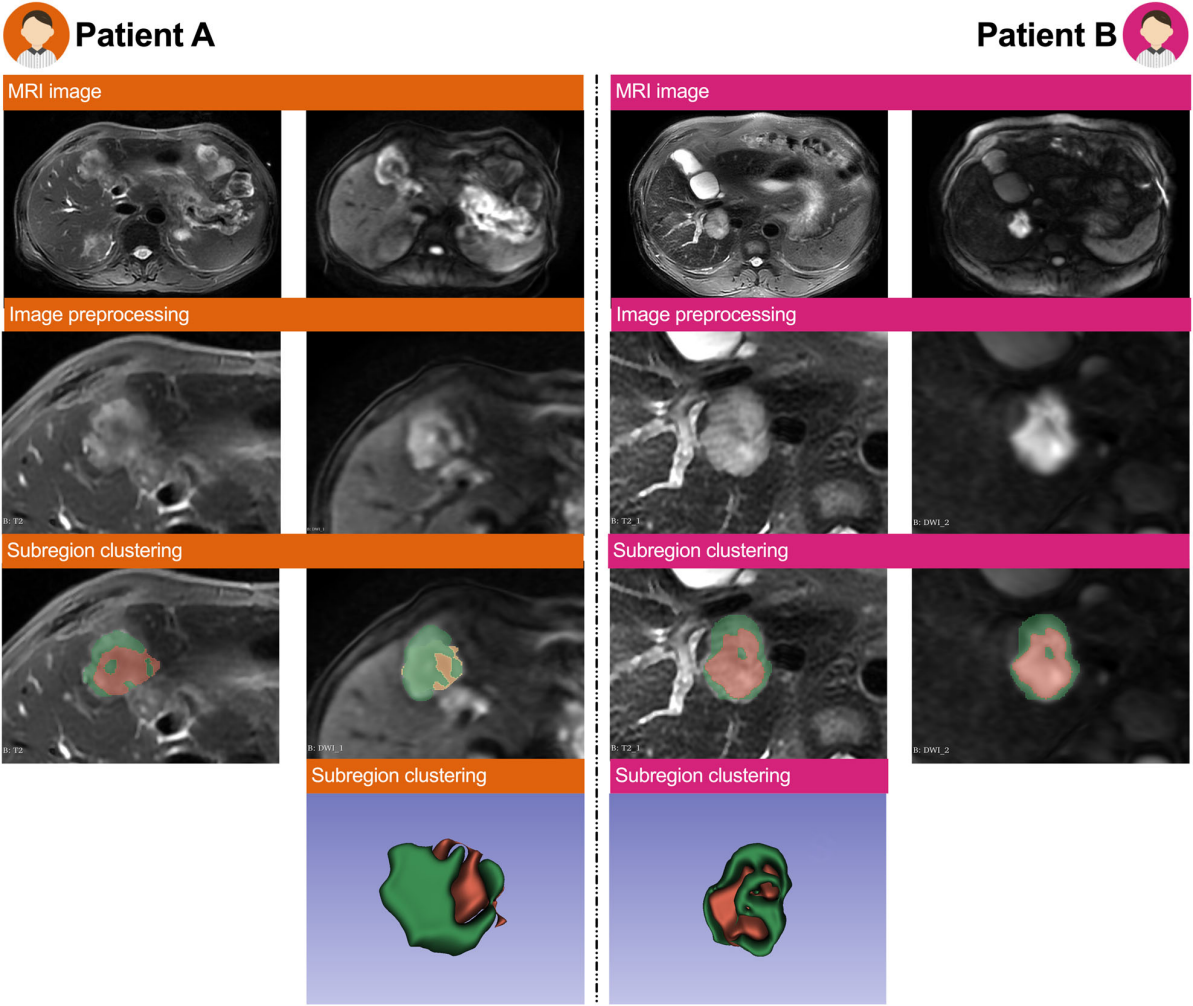
The schematic illustrates an example implementation of the Habitat clustering process. Patient A (60-year-old female, low-grade intrahepatic mass-forming cholangiocarcinoma): T2-weighted imaging demonstrated heterogeneous signal intensity with a central hypointense area and infiltrative margins. Diffusion-weighted imaging (b = 800 s/mm²) revealed marked heterogeneity, showing mixed hyperintense signal and hypointense signal. Patient B (48-year-old male, high-grade intrahepatic mass-forming cholangiocarcinoma): T2-weighted imaging exhibited homogeneous hyperintensity with well-defined borders and absence of internal necrosis. Diffusion-weighted imaging (b = 800 s/mm²) displayed heterogeneous hyperintensity accompanied by relatively regular peripheral margins

As illustrated in Fig. 3, DWI_habitat1_ represents the high-signal region of the tumor, corresponding to areas with dense tumor cells, while DWI_habitat2_ represents the low-signal region, associated with sparse tumor cells. T2WI_habitat1_ represents the high-signal region, indicating areas with higher water content in the tumor tissue, while T2WI_habitat2_ represents the low-signal region, corresponding to areas with lower water content.

To predict pathological grading, the output probabilities from the models based on the four subregions were used to generate a tumor heterogeneity index, and random combinations of these subregions were tested. A total of 13 models were developed (Table S2). Among these, the T2WI_habitat1_ + DWI_habitat2_ model achieved the highest AUC in both the training dataset (AUC = 0.847, 95% CI: 0.783, 0.911) and the internal validation dataset (AUC = 0.753, 95% CI: 0.595, 0.911). Therefore, the T2WI_habitat1_ + DWI_habitat2_ model was selected as the habitat model for further development in the combined models.

### Performance evaluation of prediction models

The diagnostic performance metrics for the seven models, including AUC, sensitivity, specificity, accuracy, and precision, are summarized in Table 3. For predicting the pathological grade of IMCC, the ClinLabImag model achieved AUCs of 0.655 (95% CI: 0.564, 0.745) in the training dataset, 0.614 (95% CI: 0.414, 0.814) in the internal validation set, and 0.675 (95% CI: 0.497, 0.853) in the external test set. The Radiomics model achieved AUCs of 0.864 (95% CI: 0.807, 0.920) in the training dataset, 0.769 (95% CI: 0.613, 0.924) in the internal validation set, and 0.736 (95% CI: 0.573, 0.900) in the external test set. Similarly, the habitat model reached AUCs of 0.847 (95% CI: 0.783, 0.911) in the training dataset, 0.753 (95% CI: 0.595, 0.911) in the internal validation set, and 0.584 (95% CI: 0.378, 0.789) in the external test set, respectively.

**Table 3:** Discrimination performance comparison of the prediction models for pathological grading

Models	AUC (95% CI)	Accuracy	Sensitivity	Specificity	Precision
ClinLabImag model					
Training cohorts	0.655 (0.564–0.745)	0.658	0.794	0.658	0.726
Internal validation cohorts	0.614 (0.414–0.814)	0.667	0.704	0.667	0.792
External validation cohorts	0.675 (0.497–0.853)	0.698	1	0.698	0.69
Radiomics model					
Training cohorts	0.864 (0.807–0.92)	0.715	0.598	0.961	0.970
Internal validation cohorts	0.769 (0.613–0.924)	0.667	0.630	0.750	0.850
External validation cohorts	0.736 (0.573–0.900)	0.698	0.690	0.714	0.833
Habitat model					
Training cohorts	0.847 (0.783–0.911)	0.658	0.561	0.863	0.896
Internal validation cohorts	0.753 (0.595–0.911)	0.667	0.593	0.833	0.889
External validation cohorts	0.584 (0.378–0.789)	0.651	0.724	0.500	0.75
ClinLabImag-radiomics model					
Training cohorts	0.876 (0.823–0.929)	0.614	0.439	0.98	0.979
Internal validation cohorts	0.778 (0.632–0.924)	0.615	0.444	1	1
External validation cohorts	0.764 (0.602–0.925)	0.744	0.897	0.429	0.765
ClinLabImag-habitat model					
Training cohorts	0.848 (0.784–0.911)	0.684	0.598	0.863	0.901
Internal validation cohorts	0.744 (0.579–0.908)	0.667	0.593	0.833	0.889
External validation cohorts	0.621 (0.426–0.816)	0.605	0.759	0.286	0.688
Radiomics-habitat model					
Training cohorts	0.891 (0.839–0.943)	0.696	0.579	0.941	0.954
Internal validation cohorts	0.799 (0.662–0.937)	0.692	0.556	1	1
External validation cohorts	0.805 (0.674–0.937)	0.767	0.828	0.643	0.828
Combined model					
Training cohorts	0.895 (0.845–0.944)	0.703	0.579	0.961	0.969
Internal validation cohorts	0.790 (0.650–0.931)	0.667	0.519	1	1
External validation cohorts	0.815 (0.680–0.951)	0.767	0.828	0.643	0.828

*CI* confidence intervals, *AUC* area under the receiver operating characteristic curve

**Fig. 4:**
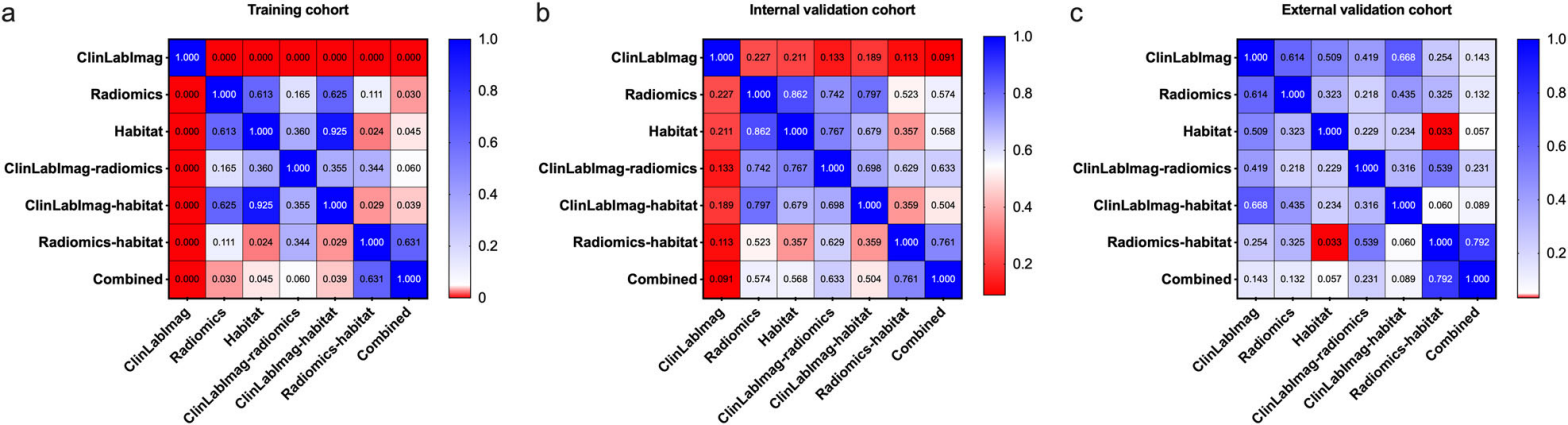
Heatmaps of DeLong test in the training cohort (**a**) internal validation cohort (**b**) and external validation cohort (**c**)

DeLong’s test (Fig. 4) revealed no statistically significant differences in AUC between the Radiomics, Habitat, and ClinLabImag models in the external validation set (*p* > 0.05 for all pairwise comparisons). However, we observed that the Radiomics model had a specificity of 0.714, which was higher than the Habitat model (0.500) and the ClinLabImag model (0.698). The Radiomics model (0.698) and ClinLabImag model (0.698) also had higher accuracy compared to the Habitat model (0.651).

**Fig. 5:**
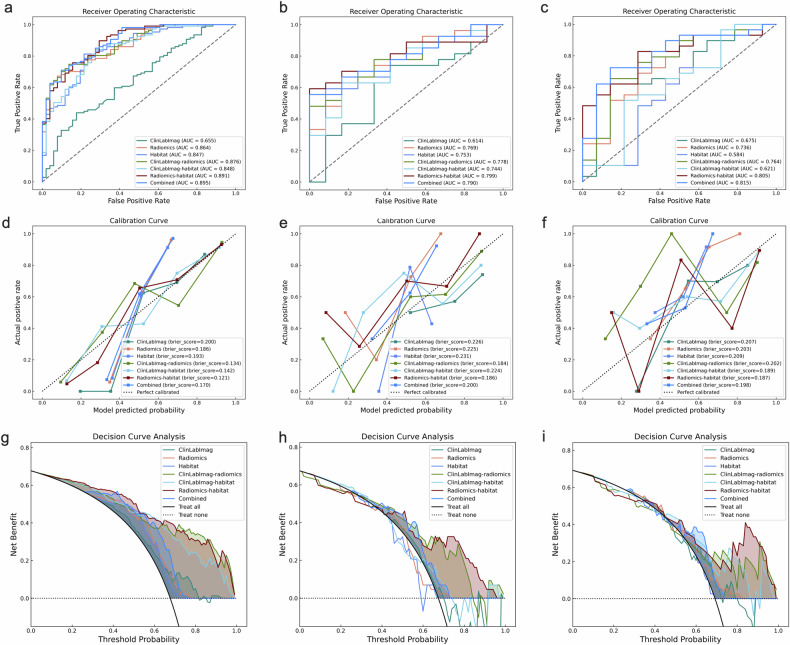
The receiver operating characteristic curves (**a**–**c**), calibration curves (**d**–**f**), and decision curves (**g**–**i**) of the different models in the training cohort, internal validation cohort and external validation cohort. AUC, area under the curve

**Fig. 6:**
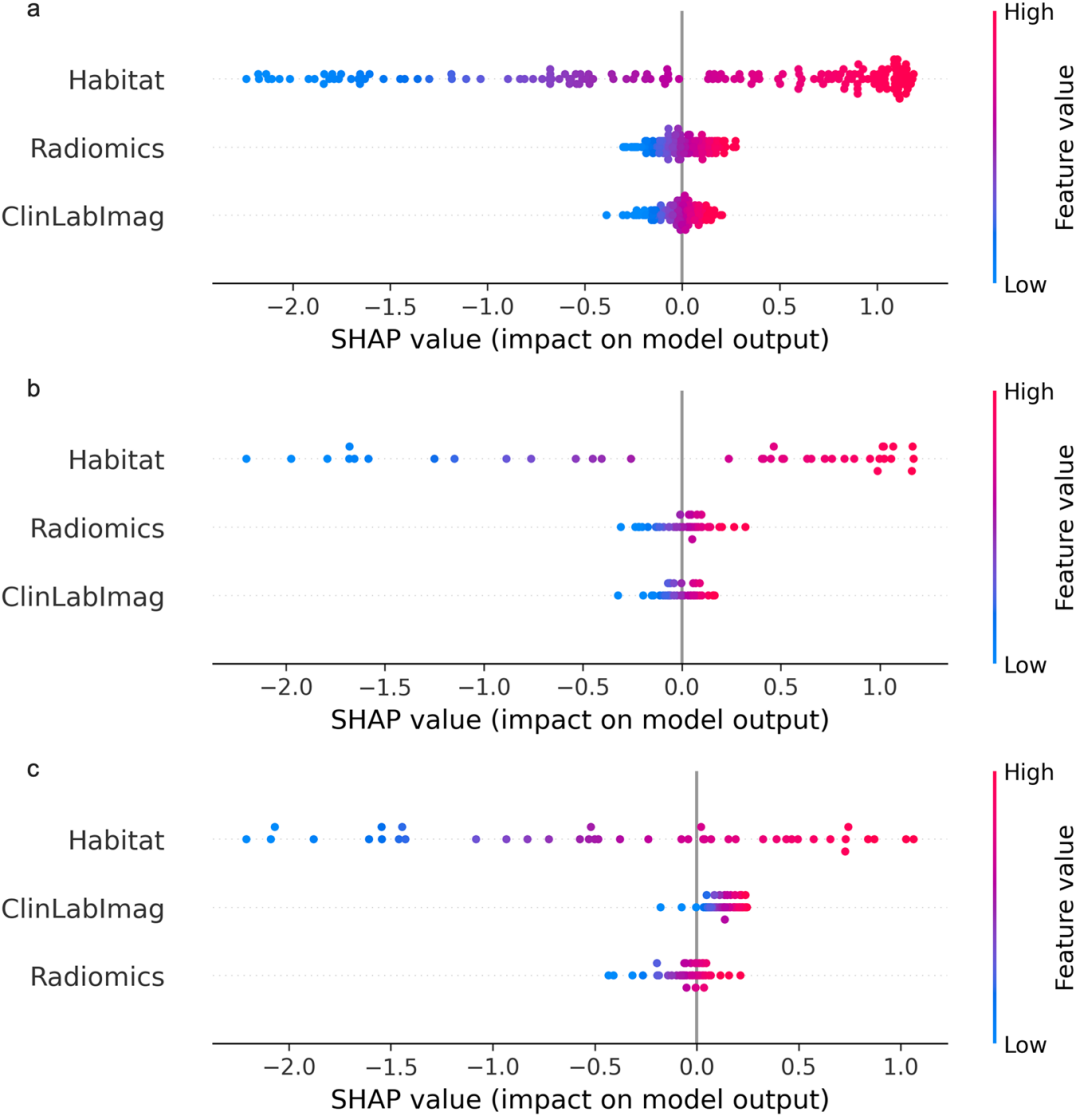
The heatmap plots show the global importance of multimodal features (clinical laboratory-imaging, radiomic, and habitat features) in the combined model across three cohorts: (**a**) training cohort, (**b**) internal validation cohort, and (**c**) external validation cohort, with bar plots displayed on the right-hand side of the heatmap. The heatmaps present the individuals on the *x*-axis, the features on the *y*-axis, and the SHAP values encoded on a color scale

Furthermore, the integration of clinical, laboratory, and imaging variables with habitat and conventional radiomics features enhanced the predictive performance of the combined model. The combined model achieved AUCs of 0.895 (95% CI: 0.845, 0.944) in the training dataset, 0.790 (95% CI: 0.650, 0.931) in the internal test set, and 0.815 (95% CI: 0.680, 0.951) in the external test set (Table 3, Fig. 5). Additionally, SHAP analysis was performed to evaluate the importance of each feature in the combined model across the training, internal validation, and external validation sets. As shown in Fig. 6, in the external validation set, the impact of the Habitat features is the most significant, with higher values (indicated in red) tending to have a positive influence on the model output. Following this, the Radiomics features exhibit a notable effect, while the ClinLabImag features demonstrate the least influence.

## Discussion

In this study, we developed a habitat model based on the quantitative ITH index derived from tumor subregions, which demonstrated good reliability and was validated in an independent external cohort. Furthermore, in the external test set, when the Habitat Model was combined with the ClinLabImag model and the Radiomics model, the resulting combined model exhibited the highest performance in predicting IMCC pathological grading, with an AUC of 0.815. Finally, SHAP analysis indicated that the quantitative ITH index was a key feature in the decision-making process of the combined model.

The SHAP analysis in this study indicated that habitat features have a significant impact on the model output, highlighting the crucial role of quantitative ITH in the model’s decision-making process. This finding may reflect the high predictive capability of subregions with different biological characteristics within the tumor [29] when predicting pathological grading, thereby emphasizing the importance of tumor subregional biological features in pathological grading prediction.

Regrettably, previous studies [20, 30] have not quantitatively explored the relationship between IMCC pathological grading and ITH. In this study, DWI_habitat2_, representing regions with low cellular density, may have captured characteristics of these sparse cell areas [28]. Additionally, T2WI_habitat1_ represents areas with higher water content, potentially correlating with specific histological features of the tumor, such as necrosis or increased mucus secretion. Moreover, previous studies [20, 28, 31] have demonstrated that the combined use of T2WI and DWI can accurately differentiate IMCC grades. These findings align with tumor grading, where high-grade tumors exhibit necrotic regions (T2WI_habitat1_) and heterogeneous cellular distribution (DWI_habitat2_), underscoring the role of MRI in quantitatively capturing biologically meaningful ITH.

Our study proposed a novel method for quantifying tumor heterogeneity, which provided critical insights for guiding treatment strategy selection. For instance, by identifying high-risk pathological grades [30], clinicians can implement targeted therapeutic interventions at an earlier stage, such as neoadjuvant chemotherapy [32] or more aggressive surgical strategies. Additionally, our model offers a non-invasive diagnostic tool [33] that has the potential to reduce reliance on traditional invasive biopsies. Given the inherent risk of sampling bias in biopsy procedures, our model can serve as a complementary approach to assist clinicians in achieving a more comprehensive preoperative assessment of tumor characteristics [34].

On the other hand, the Habitat model performed excellently in the training set (AUC: 0.847, 95% CI: 0.783, 0.911) and the internal validation set (AUC: 0.753, 95% CI: 0.595, 0.911), but its AUC significantly dropped to 0.584 (95% CI: 0.378, 0.789) in the external validation set. This decrease indicates that while habitat model based on tumor subregions may provide valuable insights into tumor biology, its generalizability might be limited by variations in imaging protocols or patient populations across centers [35, 36]. The diagnostic performance of the Habitat model lies between the Radiomics Model and the ClinLabImag model, suggesting that it indeed captures ITH features, especially in its ability to describe different subregions of the tumor (such as regions with higher water content and lower cell density) on T2WI and DWI sequences. However, the limitation of the Habitat model lies in its relatively simple feature extraction method. Compared to the Radiomics model, although the Habitat model can distinguish between different habitats within the tumor—such as areas of rich water content and low cell density—the division of these habitats is less precise in capturing heterogeneity than the Radiomics model. The Radiomics model incorporates more texture and morphological features and can even reflect molecular characteristics of the tumor, making it more detailed in capturing tumor heterogeneity [37].

In comparison to the ClinLabImag and Habitat models, the Radiomics model exhibited the highest AUC in both the training (0.864, 95% CI: 0.807–0.920) and internal validation datasets (0.769, 95% CI: 0.613–0.924), underscoring its robust predictive ability for IMCC grading. Its performance in the external test set (0.736, 95% CI: 0.573, 0.900) further reinforces its robustness and generalizability, despite a slight decline. These results align with recent literature emphasizing the potential of radiomics [30], which captures subtle nuances of intratumoral heterogeneity—a key feature for cancer grading and prognosis prediction. Such characteristics may not be fully recognized by traditional imaging and clinical laboratory data [38].

Although the ClinLabImag model integrates clinical, laboratory, and imaging features, its performance lagged behind the Radiomics and Habitat models, possibly due to its reliance on traditional imaging, clinical, and laboratory data, which have limitations in reflecting intratumoral heterogeneity [39]. Previous studies [30] that constructed predictive models for IMCC pathological grading using morphological and radiomic features reported diagnostic performance similar to the predictive models in our study, which employed the same features. Notably, the independent external validation set further supports the generalizability and reliability of our model.

### Limitations

Our study has several limitations. First, this is a retrospective analysis, which introduces potential bias in population selection, although external validation was performed to improve reliability. Further prospective analyses are warranted to explore MRI-based quantification of liver tumor heterogeneity in more detail. Second, using only DWI and T2WI does not fully capture the intratumoral heterogeneity of IMCC, which may introduce analytical bias and affect the generalizability of clinical applications. Finally, the relatively small sample size in the external validation set may limit the generalizability of our findings.

## Conclusion

In conclusion, our study demonstrates that MRI-based quantification of ITH, when combined with clinical, laboratory, radiological, and radiomics features, can improve the predictive performance of IMCC grading. By developing and validating the Habitat model, we gained deeper insights into the biological heterogeneity within the tumor and provided a novel non-invasive tool for predicting IMCC grading.

## Supplementary information

ELECTRONIC SUPPLEMENTARY MATERIAL

## Data Availability

The data used and analyzed during the current study are available from the corresponding authors on reasonable request.

## Abbreviations

AUC: Area under the receiver operating characteristic curve
DWI: Diffusion-weighted imaging
GLCM: Gray-level co-occurrence matrix
GLDM: Gray-level dependence matrix
GLRLM: Gray-level run-length matrix
GLSZM: Gray-level size zone matrix
ICC: Intrahepatic cholangiocarcinoma
IMCC: Intrahepatic mass-forming cholangiocarcinoma
ITH: Intratumoral heterogeneity
LR: Logistic regression
NGTDM: Neighboring gray-tone difference matrix
SSE: Sum of squared errors
T2WI: T2-weighted imaging
VOIs: Volumes of interest
