# Supplementary material for: MRI-based quantification of intratumoral heterogeneity for intrahepatic mass-forming cholangiocarcinoma grading: a multicenter study
Source: Insights Imaging. 2025 May 14;16:101. doi: 10.1186/s13244-025-01985-9 (PMC12078897; doi:10.1186/s13244-025-01985-9)
Supplement: Supplementary file 1 — ELECTRONIC SUPPLEMENTARY MATERIAL [file 13244_2025_1985_MOESM1_ESM.pdf]

MRI-based Quantification of Intratumoral Heterogeneity for Intrahepatic Mass-forming Cholangiocarcinoma Grading: A Multicenter Study

ELECTRONIC SUPPLEMENTARY MATERIAL

MR Image Filters

1. Additive Gaussiannoise Filter

Alter an image with additive Gaussian white noise.Additive Gaussian white noise can be modeled as:

$$I = I_0 + N$$

Where I is the observed image, I_0 is the noise-free image and $N$is a normally distributed random variable of mean σ^2 and variance :

$$N \sim N(\mu, \sigma^2)$$

The noise is independent of the pixel intensities.

2. Bilateral Filter

This filter uses bilateral filtering to blur an image using both domain and range "neighborhoods". Pixels that are close to a pixel in the image domain and similar to a pixel in the image range are used to calculate the filtered value. Two gaussian kernels (one in the image domain and one in the image range) are used to smooth the image. The result is an image that is smoothed in homogeneous regions yet has edges preserved. The result is similar to anisotropic diffusion but the implementation in non-iterative. Another benefit to bilateral filtering is that any distance metric can be used for kernel smoothing the image range. Hence, color images can be smoothed as vector images, using the CIE distances between intensity values as the similarity metric (the Gaussian kernel for the image domain is evaluated using CIE distances). A separate version of this filter will be designed for color and vector images.Bilateral filtering is capable of reducing the noise in an image by an order of magnitude while maintaining edges.

3. Binomial Blur Image Filter

Performs a separable blur on each dimension of an image.The binomial blur consists of a nearest neighbor average along each image dimension. The net result after n-iterations approaches convultion with a gaussian.

4. Box Mean Filter

Implements a fast rectangular mean filter using the accumulator approach.

5. Box Sigma Image Filter

Implements a fast rectangular sigma filter using the accumulator approach.

6. Curvature Flow Filter

Denoise an image using curvature driven flow.CurvatureFlowImageFilter implements a curvature driven image denoising algorithm. Iso-brightness contours in the grayscale input image are viewed as a level set. The level set is then evolved using a curvature-based speed function:

$$I_k =_k \backslash \nabla I \backslash$$

where κ is the curvature.

The advantage of this approach is that sharp boundaries are preserved with smoothing occurring only within a region. However, it should be noted that continuous application of this scheme will result in the eventual removal of all information as each contour shrinks to zero and disappear.

Note that unlike level set segmentation algorithms, the image to be denoised is already the level set and can be set directly as the input using the SetInput() method.

This filter has two parameters: the number of update iterations to be performed and the timestep between each update.

The timestep should be "small enough" to ensure numerical stability. Stability is guarantee when the timestep meets the CFL (Courant-Friedrichs-Levy) condition. Broadly speaking, this condition ensures that each contour does not move more than one grid position at each timestep. In the literature, the timestep is typically user specified and have to manually tuned to the application.

This filter make use of the multi-threaded finite difference solver hierarchy. Updates are computed using a Curvature Flow Function object. A zero flux Neumann boundary condition when computing derivatives near the data boundary.

This filter may be streamed. To support streaming this filter produces a padded output which takes into account edge effects. The size of the padding is m_NumberOfIterations on each edge. Users of this filter should only make use of the center valid central region.

7. Discrete Gaussian Filter

Blurs an image by separable convolution with discrete gaussian kernels. This filter performs Gaussian blurring by separable convolution of an image and a discrete Gaussian operator (kernel).The Gaussian operator used here was described by Tony Lindeberg (Discrete Scale-Space Theory and the Scale-Space Primal Sketch. Dissertation. Royal Institute of Technology, Stockholm, Sweden. May 1991.) The Gaussian kernel used here was designed so that smoothing and derivative operations commute after discretization.

8. Laplacian Sharpening Filter

This filter sharpens an image using a Laplacian. LaplacianSharpening highlights regions of rapid intensity change and therefore highlights or enhances the edges. The result is an image that appears more in focus.

9. Mean Filter

Applies an averaging filter to an image.Computes an image where a given pixel is the mean value of the the pixels in a neighborhood about the corresponding input pixel.A mean filter is one of the family of linear filters.

10. Median Filter

Applies a median filter to an image.Computes an image where a given pixel is the median value of the the pixels in a neighborhood about the corresponding input pixel.A median filter is one of the family of nonlinear filters. It is used to smooth an image without being biased by outliers or shot noise.

11. Normalize Filter

Normalize an image by setting its mean to zero and variance to one. NormalizeImageFilter shifts and scales an image so that the pixels in the image have a zero mean and unit variance. This filter uses StatisticsImageFilter to compute the mean and variance of the input and then applies ShiftScaleImageFilter to shift and scale the pixels.NB: since this filter normalizes the data to lie within -1 to 1, integral types will produce an image that DOES NOT HAVE a unit variance.

12. Recursive Gaussian Filter

Base class for computing IIR convolution with an approximation of a Gaussian kernel.

$$\frac{1}{\sigma\sqrt{2\pi}} exp(-\frac{x^2}{2\sigma^2})$$

RecursiveGaussianImageFilter is the base class for recursive filters that approximate convolution with the Gaussian kernel. This class implements the recursive filtering method proposed by R.Deriche in IEEE-PAMI Vol.12, No.1, January 1990, pp 78-87, "Fast Algorithms for Low-Level Vision"

13. Shot Noise Filter

Alter an image with shot noise.The shot noise follows a Poisson distribution:

$$I = N(I_0)$$

where $N(I_0)$ is a Poisson-distributed random variable of mean I_0. The noise is thus dependent on the pixel intensities in the image.

The intensities in the image can be scaled by a user provided value to map pixel values to the actual number of particles. The scaling can be seen as the inverse of the gain used during the acquisition. The noisy signal is then scaled back to its input intensity range:

$$I = \frac{N(I_0 \times \delta)}{\delta}$$

where δ is the scale factor.

The Poisson-distributed variable λ is computed by using the algorithm:

$$\begin{gathered} k \leftarrow 0 \\ p \leftarrow 1 \\ \text{repeat}\begin{cases} k = k+1 \\ p = p * U() \end{cases} \\ until p > e^{\lambda}, reture(k) \end{gathered}$$

where $U()$ provides a uniformly distributed random variable in the interval [0,1] .

This algorithm is very inefficient for large values of λ , though. Fortunately, the Poisson distribution can be accurately approximated by a Gaussian distribution of mean and variance λ when λ is large enough. In this implementation, this value is considered to be 50. This leads to the faster algorithm:

$$\lambda + \sqrt{\lambda} \times N()$$

where $N()$ is a normally distributed random variable of mean 0 and variance 1.

14. Smoothing Recursive Gaussian Filter

Computes the smoothing of an image by convolution with the Gaussian kernels implemented as IIR filters.This filter is implemented using the recursive gaussian filters. For multi-component images, the filter works on each component independently.For this filter to be able to run in-place the input and output image types need to be the same and/or the same type as the RealImageType.

15. Speckle Noise Filter

Alter an image with speckle (multiplicative) noise.The speckle noise follows a gamma distribution of mean 1 and standard deviation provided by the user. The noise is proportional to the pixel intensity.

It can be modeled as:

$$I = I_0 * G$$

where $G$is a is a gamma distributed random variable of mean 1 and variance proportional to the noise level:

$$G \sim \Gamma(\frac{1}{\sigma^2}, \sigma^2)$$

16. WaveletFilter

A wavelet is a wave-like oscillation with an amplitude that begins at zero, increases, and then decreases back to zero. It can typically be visualized as a "brief oscillation" like one recorded by a seismograph or heart monitor. Generally, wavelets are intentionally crafted to have specific properties that make them useful for signal processing. Using a "reverse, shift, multiply and integrate" technique called convolution, wavelets can be combined with known portions of a damaged signal to extract information from the unknown portions.

For example, a wavelet could be created to have a frequency of Middle C and a short duration of roughly a 32nd note. If this wavelet were to be convolved with a signal created from the recording of a song, then the resulting signal would be useful for determining when the Middle C note was being played in the song. Mathematically, the wavelet will correlate with the signal if the unknown signal contains information of similar frequency. This concept of correlation is at the core of many practical applications of wavelet theory.

As a mathematical tool, wavelets can be used to extract information from many different kinds of data, including – but not limited to – audio signals and images. Sets of wavelets are generally needed to analyze data fully. A set of "complementary" wavelets will decompose data without gaps or overlap so that the decomposition process is mathematically reversible. Thus, sets of complementary wavelets are useful in wavelet based compression/decompression algorithms where it is desirable to recover the original information with minimal loss.

In formal terms, this representation is a wavelet series representation of a square-integrable function with respect to either a complete, orthonormal set of basis functions, or an overcomplete set or frame of a vector space, for the Hilbert space of square integrable functions. This is accomplished through coherent states.

Table S1 MRI Acquisition Parameters for the Training and Test Datasets

Dataset	Scanner	Sequence	TR/ TE (ms)	FOV (mm)	Matrix	Sections Thickness (mm)	Section Gap (mm)	No. of Sections	Flip Angle
Training and internal test dataset	GE 3.0T (Discovery MR 750)	T2WI	(5000-8000)/(86-100)	(400, 300)	(320, 320)	5	2.5	2	120
		DWI	(3000-7500)/(46-66)	(380, 300)	(128, 130)	5	1	1	90
	UI 1.5T (uMR 588)	T2WI	(4500-6700)/(80-90)	(350, 350)	(320, 320)	5	2	2	150
		DWI	(3085-7060)/(45-70)	(380, 300)	(128, 128)	6	1	1	90
	Siemens 1.5T (Amira)	T2WI	(4099-5187)/(89-102)	(350, 350)	(320, 288)	5	2	2	100
		DWI	(3120-7000)/(45-70)	(380, 300)	(380, 250)	6	1	1	90
External test	GE 1.5T (Optima	T2WI	(4099-5187)/(89-102)	(350, 350)	(288, 288)	5	2	1	150, 120

dataset	MR360)								
		DWI	(3120-7000)/(45-70)	(380, 300)	(140, 140)	6	1	1	90
	GE 3.0T (Signa HDx)	T2WI	(3000-6000)/(67.6-85)	(350, 350)	(320, 224)	5-6	2	0.5	100,145
		DWI	(7100-9230)/(66-79.6)	(400, 320)	(128, 128)	6	1	1	90

FOV, field of view, TR, repetition time, TE, echo time, No., number, ms, millisecond, mm, millimeter

Table S2 Comparison of Pathological Grading Prediction Models Based on Subregions and Combinations

Model	Task	AUC	Accuracy	Sensitivity	Specificity	Precision
$DWI_{habitat1}$	Training Cohorts	0.826(0.756-0.895)	0.690	0.654	0.765	0.854
	Internal validation cohorts	0.608(0.419-0.797)	0.590	0.481	0.833	0.867
$DWI_{habitat2}$	Training Cohorts	0.778(0.702-0.853)	0.722	0.907	0.333	0.740
	Internal validation cohorts	0.664(0.473-0.854)	0.718	0.852	0.417	0.767
$T2WI_{habitat1}$	Training Cohorts	0.793(0.717-0.869)	0.703	0.701	0.706	0.833
	Internal validation cohorts	0.735(0.578-0.891)	0.692	0.63	0.833	0.895
$T2WI_{habitat2}$	Training Cohorts	0.784(0.712-0.856)	0.652	0.542	0.882	0.906
	Internal validation cohorts	0.583(0.378-0.788)	0.564	0.519	0.667	0.778
$DWI_{habitat1}$+$DWI_{habitat2}$+$T2WI_{habitat1}$	Training Cohorts	0.872(0.813-0.93)	0.658	0.551	0.882	0.908
	Internal validation cohorts	0.716(0.548-0.884)	0.667	0.556	0.917	0.938
$DWI_{habitat1}$+$DWI_{habitat2}$+$T2WI_{habitat2}$	Training Cohorts	0.870(0.812-0.927)	0.665	0.523	0.961	0.966
	Internal validation cohorts	0.747(0.592-0.902)	0.615	0.444	1	1
$DWI_{habitat2}$+$T2WI_{habitat1}$+$T2WI_{habitat2}$	Training Cohorts	0.891(0.841-0.942)	0.804	0.832	0.745	0.873
	Internal validation cohorts	0.639(0.453-0.825)	0.692	0.778	0.500	0.778
**$T2WI_{habitat1}$+$DWI_{habitat2}$**	Training Cohorts	**0.847(0.783-0.911)**	**0.658**	**0.561**	**0.863**	**0.896**
	Internal validation cohorts	**0.753(0.595-0.911)**	**0.667**	**0.593**	**0.833**	**0.889**
$T2WI_{habitat1}$+$DWI_{habitat1}$	Training Cohorts	0.838(0.77-0.906)	0.551	0.346	0.980	0.974
	Internal validation cohorts	0.67(0.491-0.848)	0.615	0.481	0.917	0.929
$T2WI_{habitat1}$+$T2WI_{habitat2}$	Training Cohorts	0.842(0.777-0.907)	0.671	0.589	0.843	0.887
	Internal validation cohorts	0.722(0.549-0.896)	0.667	0.556	0.917	0.938
$DWI_{habitat1}$+$DWI_{habitat2}$	Training Cohorts	0.864(0.806-0.921)	0.589	0.402	0.98	0.977
	Internal validation cohorts	0.642(0.454-0.83)	0.513	0.338	0.917	0.900

T2WI$_{habitat2}$+DWI$_{habitat2}$	Training Cohorts	0.840(0.779-0.901)	0.715	0.720	0.706	0.837
	Internal validation cohorts	0.639(0.443-0.835)	0.615	0.667	0.500	0.750
T2WI$_{habitat2}$+DWI$_{habitat1}$	Training Cohorts	0.870(0.811-0.929)	0.658	0.542	0.902	0.921
	Internal validation cohorts	0.617(0.424-0.811)	0.590	0.481	0.833	0.867
Whole	Training Cohorts	0.889(0.836-0.943)	0.671	0.533	0.961	0.966
	Internal validation cohorts	0.719(0.557-0.881)	0.641	0.519	0.917	0.933

AUC, area under the receiver operating characteristic curve.
